# Influence of opioid-related side effects on disability, mood, and opioid misuse risk among patients with chronic pain in primary care

**DOI:** 10.1097/PR9.0000000000000589

**Published:** 2017-03-06

**Authors:** Robert N. Jamison, Kathleen Dorado, Anna Mei, Robert R. Edwards, Marc O. Martel

**Affiliations:** a Pain Management Center, Department of Anesthesiology, Brigham and Women's Hospital, Harvard Medical School, Boston, MA, USA; b Faculties of Dentistry and Medicine, McGill University, Montreal, Canada

**Keywords:** Side effects, Opioids, Primary care, Pain management, Chronic pain, Side effects, Medication misuse

## Abstract

**Background:**

There is increasing concern among primary care practitioners about the use of opioids for chronic pain, including their adverse effects, but little attention has been given to how reports of side effects from prescription medication can contribute to outcomes among patients with chronic pain. The aim of this study was to investigate the impact of frequently reported side effects on mood, disability, and opioid misuse in patients with chronic pain prescribed opioids within primary care.

**Methods:**

Two hundred (N = 200) patients with chronic pain taking opioids for pain were recruited into the study. All patients completed baseline measures and a monthly side effects checklist once a month for 6 months. Patients were divided evenly based on a median split of the number of endorsed side effects over 6 months. The subjects repeated the baseline measures at the end of the study period.

**Results:**

Over time, reports of medication side effects tended to decrease, but differences in frequency of reported side effects from baseline to follow-up (6-month time) were not significant, and the order of the frequency of the reported side effects remained similar. Patients who reported significant medication-related adverse effects reported significantly greater activity interference, negative affect, and catastrophizing compared with those with fewer side effects (*P* < 0.01). In addition, those patients with pain who reported more side effects showed significantly higher scores on opioid misuse risk (*P* < 0.001).

**Discussion:**

This study demonstrates the important role of monitoring medication-related side effects among patients with chronic pain who are prescribed opioid medication for pain within primary care.

## Abstract 2

Patients who reported significant medication-related side effects reported greater activity interference, negative affect, catastrophizing, and opioid misuse risk compared with those with fewer side effects.

## 1. Introduction

Chronic pain is a significant public health–related problem that negatively impacts quality of life among millions of individuals and costs society an estimated $560 to $635 billion annually.^21^ Primary care providers often face challenges related to undertreated pain and the need to prescribe opioids for their patients who report severe debilitating pain. However, there are also ongoing concerns about misuse, abuse, and addiction related to use of opioids for chronic noncancer pain. With increased availability of opioids over the past decade, prescription drug abuse has been perceived to be a fast-growing problem,^13^ and the co-occurrence of prescription opioid misuse and chronic pain has become very common.^10,32^ Fear of regulatory scrutiny about improper prescribing practices associated with opioids is also a primary concern among prescribing physicians. This has resulted in tension between the need to treat legitimate medical conditions, such as postoperative pain and failed surgery, while also minimizing the risk of opioid abuse and addiction. According to provider survey studies, more than one-half of physicians surveyed reported that they were worried about scrutiny regarding opioid prescribing,^19^ and potential professional sanctions.^27^ The safe use of opioids for treatment of chronic pain has become a particular concern among health care providers and a high priority had been place on the identification of those who are at the greatest risk of opioid misuse.^25^

All prescription medications have the potential for associated side effects, and the monitoring of side effects is clinically important. Ingesting medications can add to risk of unintended consequences, which can be mild without any long-term effects (eg, fatigue) or, in some situations, quite severe (eg, gastrointestinal bleeding, heart, and liver disease). Although certain medication side effects may have perceived beneficial qualities (eg, weight loss), most are unwanted and can interfere with overall quality of life including headaches, nausea, dizziness, constipation, and weakness. Also, some medications, that are proven to be helpful in treating a certain medical condition, are discontinued because they cannot be tolerated because of their side effects. Studies have examined the effect of medication-related side effects among patients with chronic pain on treatment satisfaction,^16,18,28^ but few studies have investigated the influence of medication side effects on pain-related outcomes among patients with chronic pain. The bulk of previous studies conducted in this area primarily examined the influence of medication side effects on patients' daily functioning or quality of life. Martel et al.^29^ showed that medication side effects were associated with heightened pain-related activity interference among patients treated in specialized, tertiary care pain clinics. To our knowledge, such studies have yet to be conducted among patients with pain seen in primary care settings.

The aim of our study was to extend previous work by exploring the influence of medication side effects on mood disorder, negative affect, and opioid misuse among primary care patients with chronic pain. We hypothesized that patients who reported experiencing many medication-related side effects would show greater pain intensity and pain-related disability, higher emotional distress, and possibly higher risk of opioid misuse compared with those who reported fewer medication-related side effects.

## 2. Methods

This study was approved by the institutional review boards (IRBs) of each of the participating health care organizations and centers, and informed consent was obtained from all the participants before any study procedure.

We identified and recruited patients who had a diagnosis of chronic pain from primary care centers located within the Metropolitan Boston area as part of a larger study.^27^ Subjects were included if they (1) had chronic pain for >6 months' duration, (2) averaged 4 or greater on a pain intensity scale of 0 to 10, (3) were able to speak and understand English, (4) had been prescribed or were eligible to be prescribed opioid therapy for pain based on the practitioner's personal criteria for eligibility for chronic opioid therapy, and (5) were under the care of a primary care physician.

Patients were excluded from participation if they meet any of the following criteria: (1) diagnosis of cancer or any other malignant disease, (2) acute osteomyelitis or acute bone disease, (3) present or past DSM-IV diagnosis of schizophrenia, delusional disorder, psychotic disorder, or dissociative disorder that would be judged to interfere with study participation, (4) pregnancy, (5) any clinically unstable systemic illness judged to interfere with treatment, (6) a pain condition requiring urgent surgery, and (7) an active substance use disorder, such as cocaine or IV heroin use (positive on the MINI International Neuropsychiatric Interview; M.I.N.I. v.5.0),^26^ that would interfere with study participation.

Patients were recruited through flyers placed in the centers and were invited to participate by their treating physicians. Patients were followed up with a letter describing the study and an informed consent form that they were instructed to complete and mail back to the research assistant assigned to this study. All patients received a $50 gift card for completing the packet of questionnaires at the start of the study. Patients were informed that they would be called each month to obtain information about their health status over 6 months, to track their medication use, and to determine the presence of any side effects related to their medication. They were informed that information from the study would be shared with their practitioners, but that their information was protected through the guidelines of a confidentiality certificate through the National Institutes of Health (NIH; www.nidcr.nih.gov) as a research participant and that personal identifiable information would be protected from disclosure.

### 2.1. Baseline measures

After providing consent, patients were asked to complete the following self-report questionnaires:

#### 2.1.1. Demographic questionnaire

This baseline questionnaire collected basic demographic information about the patients, including age, sex, racial background, education level, marital status, history of medical problems, history of psychiatric treatment, and disability or worker's compensation status.

#### 2.1.2. Side effects checklist

This 12-item checklist was adopted from the original 20-item side effects checklist (SEC)^22^ and created based on the most frequently described adverse effects of opioids. All participants were asked whether they had experienced any of the following 12 side effects over the past month: constipation, dizziness, dry mouth, headache, itching, confusion, nausea, nightmares, sneezing, sweating, visual problems, weakness, or any other unnamed side effects. Patients were not asked to provide ratings of medication side effects based on a specific medication, but rather based on all the medications that they were taking. The SEC is similar to other self-report measures of medication side effects that have been used in previous studies.^20,28^

#### 2.1.3. The Brief Pain Inventory

The Brief Pain Inventory (BPI) is a well-known measure of clinical pain.^11^ The questionnaire provides information about pain history, intensity, and location as well as the degree to which the pain interferes with daily activities, mood, and enjoyment of life. Scales (rated from 0 to 10) indicate the intensity of pain in general, at its worst, at its least, and pain “right now.” Test–retest reliability for the BPI reveals correlations of 0.93 for worst pain, 0.78 for usual pain, and 0.59 for pain now. Research suggests that the BPI has adequate validity.^11^

#### 2.1.4. The Pain Disability Index

This inventory consists of 7 questions designed to measure the degree to which patients believe that their pain interferes with their functioning in family and home responsibilities, recreation, social activities, occupation, sexual behavior, self-care, and life-support (eating and sleeping) activity.^35^ Patients respond to each item on 0- to 10-point scales anchored with descriptors ranging from “no disability” to “total disability.” This measure has adequate internal consistency (Cronbach alpha = 0.86) and test–retest reliability (0.91) and is a valid measure of disability.^35^

#### 2.1.5. The Hospital Anxiety and Depression Scale

The Hospital Anxiety and Depression Scale (HADS) is a 14-item scale designed to assess the presence and severity of anxious and depressive symptoms.^37^ Seven items assess anxiety, and 7 items measure depression, each coded from 0 to 3. The HADS has been used extensively in clinics and has adequate reliability (Cronbach Alpha = 0.83) and validity, with optimal balance between sensitivity and specificity.^2^ It has been translated into many languages and is widely used around the world in clinical and research settings.

#### 2.1.6. Pain Catastrophizing Scale

The Pain Catastrophizing Scale (PCS) is a 13-item instrument that examines 3 components of catastrophizing: rumination, magnification, and helplessness. The PCS is found to predict levels of pain and distress among clinical patients, and scores have been related to thought intrusions.^33,34^ It has good psychometric properties with adequate reliability and validity and is associated with levels of pain, depression, and anxiety.

#### 2.1.7. Screener and Opioid Assessment for Patients with Pain-Revised

The Screener and Opioid Assessment for Patients with Pain-Revised (SOAPP-R) is a 24-item, cross-validated, self-administered screening instrument revised from the original SOAPP v.1^6,7^ used to help determine risk potential for aberrant drug-related behavior. Items are rated from 0 = never to 4 = very often, and their sum is the total SOAPP-R score. The SOAPP-R has been shown to have good predictive validity, with an area under the curve ratio of 0.88 (95% confidence interval [CI], 0.81–0.95). Test–retest reliability was 0.71 with a coefficient alpha of 0.74. A cutoff score of 18 shows adequate sensitivity (0.86) and specificity (0.73) and has been cross-validated.^4^

### 2.2. Follow-up measures

Each month, patients were called and asked to complete the same items from the SEC over the phone. After 6 months, all subjects were asked to repeat the baseline measures of the BPI, HADS, Pain Disability Index and the *Current Opioid Misuse Measure* (*COMM*).^5^ The COMM is a 17-item self-reported questionnaire designed to assess current aberrant medication-related behaviors during opioid treatment. All items are rated from 0 = never to 4 = very often, with a total maximum score of 68. Construct validity has been shown to be adequate, with positive correlates with urine toxicology results (*P* < 0.05). Test–retest reliability was 0.86 with a 95% CI ranging from 0.77 to 0.92. The overall accuracy of the COMM for predicting current aberrant drug-related behavior, as measured by the area under the curve ratio, was 0.81 (95% CI, 0.74–0.86; *P* < 0.001), and coefficient α (0.86) for the 17 items suggests adequate reliability. A cutoff score of 8 yielded a sensitivity of 0.75 and specificity of 0.65.

Patients were paid $50 once the completed follow-up packet of questionnaires was received. Packets were resent if they were not received within a month of the mailing, and all patients were called to verify that they had received the second packet of questionnaires.

### 2.3. Statistical analyses

Analyses were conducted using SPSS (IBM, version 22.0). Descriptive data for continuous variables were presented as mean values and SDs, and data for categorical variables were presented as percentages. Total scores and average values were calculated for the baseline measures and follow-up measures. Both parametric (*t* test) and nonparametric analyses (χ^2^ or Wilcoxon Signed Rank tests) were used to compare results depending on the variables, with 2-sided *P* values set at *P* < 0.05.

## 3. Results

### 3.1. Descriptive data

Altogether, 12 primary care centers and 200 patients with chronic pain located within the metropolitan Boston area were recruited for this study. Of all the patients (N = 200), 60.0% were women, and most were Caucasian (72.4%). Although most subjects had back or neck pain, only 8 percent reported having low back pain alone. A great majority of the patients (74.5%) reported having multiple pain sites. One hundred ninety-four (97.0%) of the patients reported taking prescription opioids at the start of the study. Six patients, who were initially not taking prescription opioids, were prescribed opioids for pain during the 6-month study trial. Of those patients prescribed opioids at baseline, 59.2% were prescribed short-acting opioids alone (oxycodone 60.1%, hydrocodone 23.0%, hydromorphone 10.8%, morphine 3.4%, codeine 2.7%), 16.0% were prescribed extended-release, long-acting opioids alone (methadone 35.2%, oxycodone 28.1%, morphine 25.4%, and transdermal fentanyl 11.3%), 24.5% were prescribed both short-acting and extended-release, long-acting opioids, 7.5% were prescribed tramadol, and 2.1% were prescribed buprenorphine and naloxone alone. Other nonopioid prescription medications and the number of subjects taking them included anticonvulsants (14.5%; N = 29), nonsteroidal anti-inflammatory drugs (6.0%; N = 12), antidepressants (5.0%; 5.0%), muscle relaxants (4.0%; N = 8), and anxiolytics and sedatives (2.0%; N = 4). Four subjects were using lidocaine, one was using benadryl, and one was taking Marinol. We did not track intake of nonprescription medication.

The frequency of reported medication side effects was averaged, for each patient, across the 6-month period. All patients were then grouped as “high” or “low” in medication side effects based on a median split (median = 2.33). The mean number of side effects was 2.85 (±2.33, range 0.0–11.33) over a 6-month period. Patients were divided based on a median split, and those reporting less than an average of 2.33 side effects were classified as having few side effects (N = 99, mean = 1.00 ± 0.71, median = 1.00, range 0.0–2.25), and those reporting equal to or greater than 2.33 side effects each month were classified as having many side effects (N = 101, mean = 4.66 ± 1.90, median 4.17, range 2.33–11.33). As can be seen from Table 1, adverse effects associated with prescription medication were identified with the highest being dry mouth (49.0%), constipation (36.7%), sweating (31.1%), itching (28.1%), headache (27.6%), and weakness (27.6%).

**Table 1:**
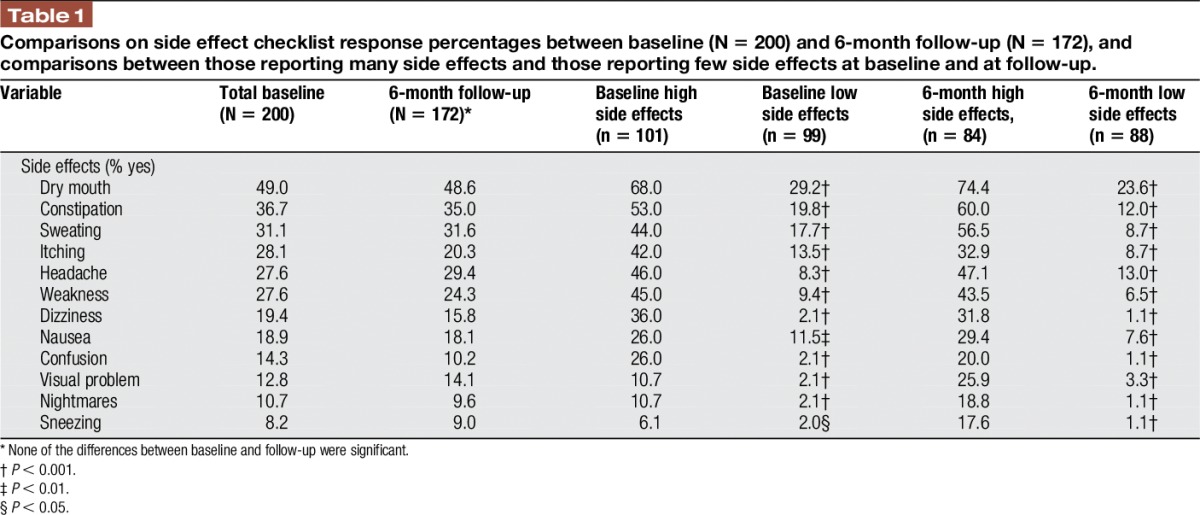
Comparisons on side effect checklist response percentages between baseline (N = 200) and 6-month follow-up (N = 172), and comparisons between those reporting many side effects and those reporting few side effects at baseline and at follow-up.

Over time, reports of medication side effects tended to decrease, but differences in frequency of reported side effects from baseline to follow-up (ie, 6-month time point) were not significant. Dry mouth, constipation, sweating, headache, and weakness continued to be associated with taking prescription medication. The order of the frequency of the reported side effects remained similar between those with many side effects and those with few side effects, although those classified as having few side effects reported experiencing weakness, headache, and dizziness much less often.

### 3.2. Correlates of medication side effects at baseline

Patients with “high” and “low” medication side effects did not differ significantly in terms of age, sex, ethnicity, pain site, pain intensity, and pain relief (Table 2). However, patients reporting high medication side effects scored significantly higher on measures of activity interference (*P* < 0.05), disability (*P* < 0.05), catastrophizing (*P* < 0.01), and on the HADS anxiety subscale (*P* < 0.05) compared with those with low side effects. Patients with a higher frequency of self-reported side effects also scored significantly higher on the SOAPP-R (*P* < 0.001; Table 3).

**Table 2:**
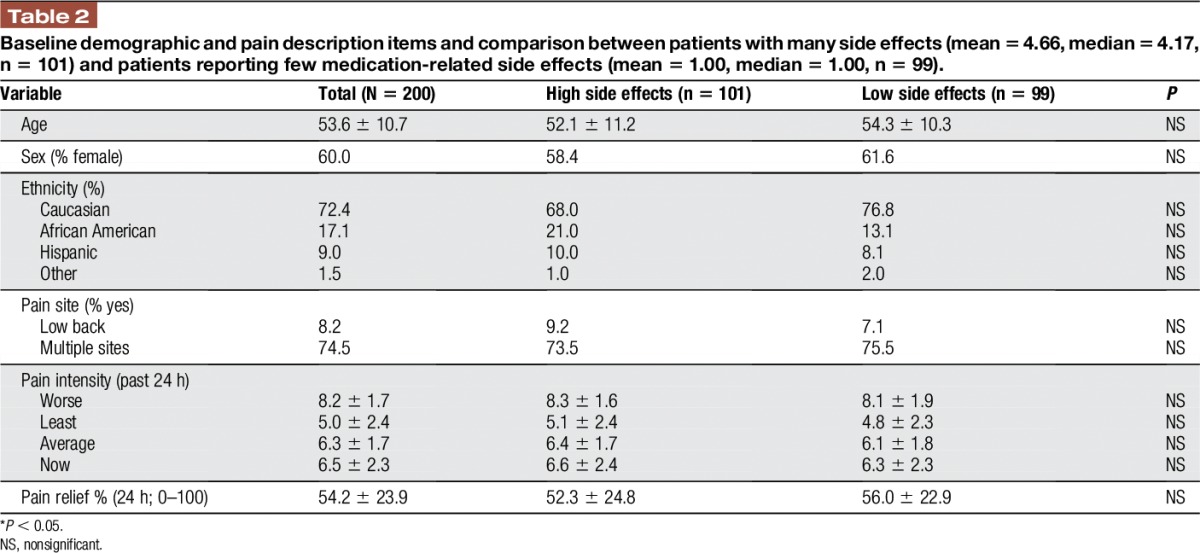
Baseline demographic and pain description items and comparison between patients with many side effects (mean = 4.66, median = 4.17, n = 101) and patients reporting few medication-related side effects (mean = 1.00, median = 1.00, n = 99).

**Table 3:**
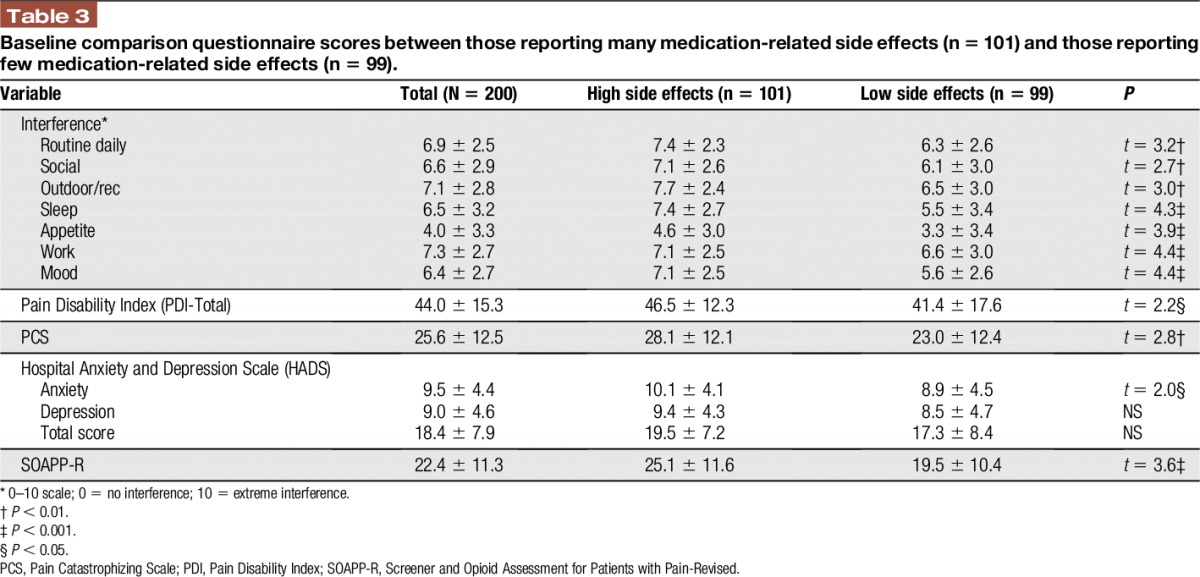
Baseline comparison questionnaire scores between those reporting many medication-related side effects (n = 101) and those reporting few medication-related side effects (n = 99).

### 3.3. Correlates of medication side effects over time

Based on the telephone interview data at 6 months after start of the study, average pain scores among all the subjects remained relatively constant (before = 7.0 ± 1.9, after = 6.7 ± 2.0), and most continued to report above average activity interference (daily routine before = 6.8 ± 2.6/10, after = 6.7 ± 2.5/10). At 6-month follow-up, those patients with chronic pain who reported a higher number of average side effects again tended to report significantly more activity interference compared with those who reported a lower number of side effects (Table 4). Significant differences were also found between those patients with chronic pain classified as experiencing few side effects compared with those reporting many side effects with higher scores among those with a higher number of side effects on mood (HADS total *P* < 0.01), pain catastrophizing (*P* < 0.001), disability (Pain Disability Index *P* < 0.001), and opioid misuse (COMM; *P* < 0.001; Table 4).

**Table 4:**
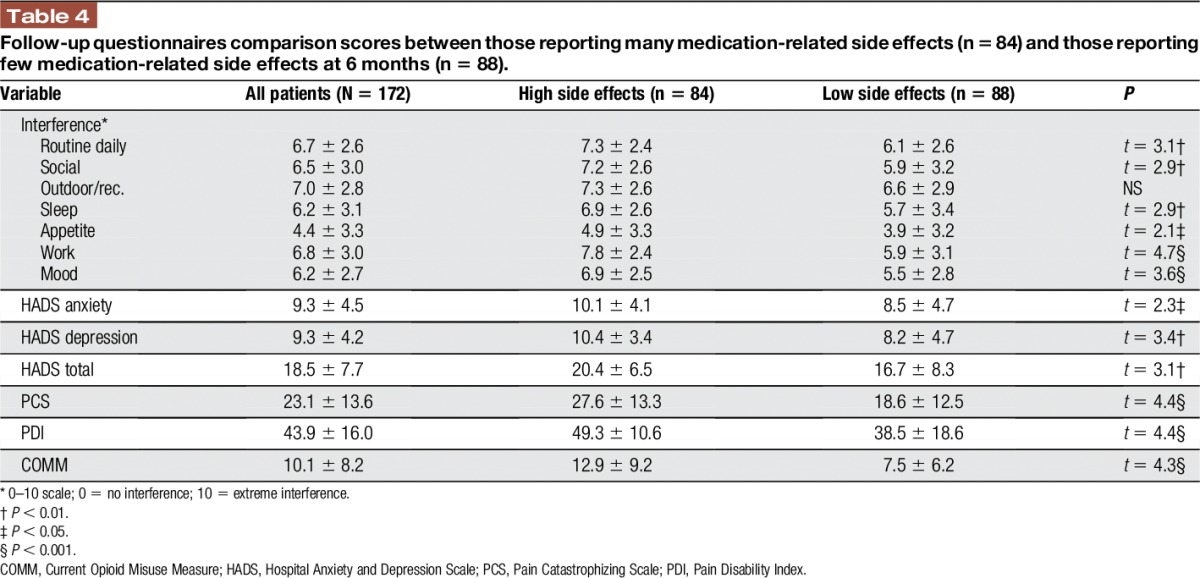
Follow-up questionnaires comparison scores between those reporting many medication-related side effects (n = 84) and those reporting few medication-related side effects at 6 months (n = 88).

## 4. Discussion

This prospective secondary data analyses study was designed to determine how reports of medication-related side effects over 6 months were associated with disability, mood, and opioid misuse among patients with chronic pain on opioid therapy within primary care. Our main findings showed that patients who reported experiencing many side effects from their medication showed greater activity interference, negative affect, and greater catastrophizing than those reporting a lower number of side effects. Another important finding of this study was that patients who were more prone to misuse opioids reported more adverse effects of the medication. It might be predicted that those who would overuse or misuse opioids would report fewer side effects of opioids because they would like taking opioids, which is in agreement with past studies that show that opioid liking predicted misuse.^3,30^ However, all patients in this study had been taking opioids for over 6 months and would likely not report any euphoria related to taking pain medication. Instead, these findings seem to support an association of sensitivity to medications with functional limitations, mood disorder, and opioid misuse. These results also emphasize the importance of attending to patient reports of adverse effects from taking prescription medication as a prediction of future problematic outcomes. These findings are in agreement with studies using quantitative sensory testing which show that those who are hyperalgesic also are prone to misuse opioids.^15^ This study suggests that those with opioid misuse risk are sensitive to reactions of medications as well.

Most prescription medications have associated adverse effects, and it is common for users of opioids to report dry mouth, constipation, and itching, among other symptoms. However, these findings have clinical implications for assessment and treatment of patients with chronic pain in primary care. In agreement with findings among patients in specialty pain centers,^29^ those who report more medication-related side effects present with a disposition toward future problems. Patients with pain who have a significant mood disorder, who catastrophize, and who are more disabled due to their pain tend to ruminate about pain and may tend to focus on symptoms and experience helplessness when in pain. There is evidence that patients who demonstrate the most emotional distress are prone to be prescribed opioids for pain.^8,24^ It should also be pointed out that despite taking opioids for pain, many of the patients reported significant pain and disability. Past research indicates that those with greater negative affect tend not to benefit from opioids.^23,36^ Thus, the long-term benefit of prescribing opioid therapy for any patient who presents with high levels of medication-related side effects should be examined. However, contrary to our hypothesis, no differences were found in pain intensity between those who reported experiencing more medication-related side effects and those with fewer side effects. Thus, reports of side effects are not just related to reports of general hypersensitivity. Future studies are needed to help understand this negative finding.

It is interesting that, in general, those who scored higher on the PCS reported a higher incidence of adverse effects to prescription medication. By definition, those who catastrophize and have recurrent worried thoughts tend to focus on symptoms and be hypervigilant to new sensations. Whether the higher incidence of reported side effects was related to heightened attention to physical symptoms or whether persons who tend to catastrophize have a physical predisposition that may make them more susceptible to adverse effects of medication needs to be addressed in future investigations. Because those who catastrophize have been found to be hyperalgesic based on quantitative sensory testing,^14^ there is support to suggest that these individuals may also have a heightened sensitivity to adverse reactions to medication. Future research may focus on objective assessment of differences in physical reactions to medications based on the level of catastrophizing.

There has been a recent change in attitudes among providers toward the use of chronic opioid therapy to treat noncancer pain. This has been particularly notable among younger primary care practitioners (PCPs).^24,27^ Recent guidelines have encouraged increased emphasis on educating PCPs on issues of chronic pain and substance abuse.^31^ Although opioids will continue to be offered for acute pain management, there is less willingness to sustain patients on chronic opioid therapy. Centers for Disease Control (CDC) guidelines encourage risk assessment, periodic urine screens, consultation with prescription monitoring programs, opioid agreements, and frequent monitoring of all patients prescribed opioids for pain.^9^ As this study suggests, assessment of medication-related side effects would also be valuable as a predictor of future opioid misuse.^1^ Regular adherence monitoring by clinicians may further assist in preventing physical and psychological toxicity such as drug abuse and addiction.^17^

This study has a number of limitations that should be highlighted when interpreting these results. First, we did not relate the specific side effects to the specific drugs taken for pain, and we did not collect medication dosage. It is possible that those who reported more side effects were taking higher doses of medication. We also monitored side effects as either present or absent, and did not assess the intensity of each of the side effects that were experienced. It might be shown that the severity of the side effects may have more predictive power than the total number of the side effects experienced. It is also possible that some of the reported side effects may have already been present before taking opioids, and scores on the SEC may not represent adverse reactions only to prescription medications. Most of the items on the SEC represent somatic symptoms, and this checklist may well be measuring somatic complaints. Future investigations might consider teasing out this relationship by including measures that assess multiple physical symptoms^12^ and by obtaining side effect reports before the subjects are prescribed opioids. Second, although this is a longitudinal study, we also cannot assume that the outcomes at 6 months followed a progression from baseline. Close monitoring with daily assessments might help to address this study limitation. Third, this study was conducted in an urban area in the northeast region of the United States, and it is uncertain whether these results might be affected by selection bias based on willingness to participate or by regional differences. Although some patients dropped out before completing the study, which could have contributed to selection bias, we did not find differences among those who completed the 6-month trial and those who did not. Fourth, the assessments were all self-report measures and objective measures of function or opioid compliance were not included. We did not capture the participants' duration of pain and the duration of using opioids. Although validated measures were used to assess opioid risk and misuse, the results were based on self-report. Future studies would benefit from results of urine toxicology screens and provider ratings of compliance. Also, many measures were included in the study, and it is possible that some results would be significant by chance alone. Finally, it should be noted that these results are correlational and do not assume causation.

Despite these limitations, the results of this study emphasize the importance of monitoring adverse effects of prescription pain medication and provide some insight into possible psychosocial risk factors of poor outcome in treating patients with chronic pain. This study is also notable because it is the first to demonstrate that primary care patients who report a greater number of side effects, possibly related to opioid use, may be at greater risk of medication misuse. Our study is one of the few that have been conducted to assess the relationship between opioid use, side effects, and psychosocial factors in primary care. Managing complex pain patients in primary care settings can be extremely challenging. PCPs typically have limited time, training, and resources to effectively evaluate patients with chronic pain. These findings have clinical implications that support the benefits of carefully monitoring reports of side effects of patients who are prescribed opioids in primary care centers. This might have a positive effect in reducing opioid misuse, although additional controlled trials are needed to empirically establish this effect.

## Disclosures

The authors have no conflicts of interest to declare.
